# Assessment of Intra- and Inter-observer Measurement Variability in a Radiographic Metacarpophalangeal Joint Osteophytosis Scoring System for the Horse

**DOI:** 10.3390/vetsci7020039

**Published:** 2020-04-06

**Authors:** Luca Lacitignola, Annarita Imperante, Francesco Staffieri, Rocco De Siena, Pasquale De Luca, Arianna Muci, Antonio Crovace

**Affiliations:** 1 Dipartimento dell’Emergenza e dei Trapianti di Organi (DETO), Sezione di Cliniche Veterinarie e P.A, Università degli Studi di Bari “Aldo Moro”, s.p. per Casamassima Km 3. Valenzano, 70010 Bari, Italy; francesco.staffieri@uniba.it (F.S.); antonio.crovace@uniba.it (A.C.); 2 Dottorato di ricerca in “Trapianti di Tessuti ed Organi e Terapie Cellulari”, Dipartimento dell’Emergenza e dei Trapianti di Organi (DETO), Università degli Studi di Bari “Aldo Moro”, 70010 Bari, Italy; im_anna6@outlook.com; 3 Equine practitioner, 70100 Bari, Italy; desienarocco@libero.it (R.D.S.); pasqualedeluca84@libero.it (P.D.L.); ariannamuci@live.it (A.M.)

**Keywords:** horse, metacarpophalangeal joint, radiographs, osteoarthritis, osteophytosis

## Abstract

The study evaluated the intra- and inter-observer measurement variability of an osteophytosis metacarpophalangeal joint scoring system. Ten (*n = 10*) dorso/palmar, latero/medial, and oblique views of equine metacarpophalangeal joints affected by osteoarthritis were examined. Nine assessment points were graded (scale: 0–3) twice by five veterinary students (inexperienced group, I) and four equine veterinary surgeons (expert group, E). The grades for each of the nine factors were summed to obtain the osteophytosis score. The variability between the two measurements was −2.04 ± 3.5, 95% CI −3.04 to −1.03 for the I group. For the E group, they were 0 ± 1.43, 95% CI −0.45 to 0.45. In the evaluation of the same radiographs, the I group had a coefficient of variability (CV) of 37.29%. The correlation was r = 0.90%. The CV between groups was 28.85%. The mean difference between the two observations was -0.03 ± 0.29 in the E group and 0.22 ± 0.77 in the I group. The I group showed a greater CV when the score was low (r = −0.78) compared to the E group, where the CV was independent of severity of osteophytosis (r = −0.47). The osteophytosis scoring system is an easily applicable and feasible system to be used by observers with different levels of experience, but inexpert observers may need additional training or may need to be helped by reference images. These data are validated by the low inter- and intra-observer measurement variability results in the E group. Therefore, the scoring system proposed seems to be a repeatable instrument applicable to the radiographic score of the severity of metacarpophalangeal joint osteoarthritis.

## 1. Introduction

Osteoarthritis (OA) can be diagnosed radiographically taking into consideration some alterations of the joint components caused by the pathological process.

Radiology represents the first level diagnostic imaging technique available in clinical practice. The earliest signs of joint disease may not be visible on radiograms, because the fissures in the articular cartilage and synovial hypertrophy are usually not recognized [1].

The radiology findings observed in metacarpophalangeal (MCP) osteoarthritis include alterations in periarticular soft tissues, synovial effusion, enthesopathy, intra-articular mineralization, subchondral sclerosis, formation of subchondral cysts, thinning of the joint space, and osteophytosis [2,3,4,5,6].

Joint scores facilitate comparisons between patient follow-ups and comparisons between patients in an effort to correlate joint abnormalities with clinical signs. A whole joint scoring system has been previously proposed, using second level diagnostic imaging in ex-vivo MCP joints. The author’s findings showed that MRI and CT were more sensitive than radiology, but osteophytes are interpreted similarly among observers using CR, CT, and MRI, which resemble those reported for the radiographic grading of both human and dog osteophytes [7]. In fact, periarticular osteophytes are a common finding in osteoarthritis easily detectable by radiology. Osteophytosis represents the outcome of endochondral ossification on bone margins and is an expression of the severity of OA [7,8]. The aim of the present study was to evaluate the intra-observer and inter-observer variability of a method for evaluation of MCP joint osteophytes, using a scoring scale.

## 2. Materials and Methods

### 2.1. Patient Inclusion Criteria

The medical records of the horses presented for lameness examination at the veterinary surgery unit of the section of veterinary clinics and animal production of the Department of Emergencies and Organ Transplantation (DETO) of the University of Bari (Italy), in between January and June, 2019, were examined. The orthopedic records were examined, and adult patients of any breed who, at the clinical examination, showed lameness localized to the region of the MCP joint by intraarticular joint block, were selected. Clinical cases presenting with fractures, dislocations, and other conditions different from osteoarthritic signs were discarded. Records had to include a complete radiographic examination with lateral/medial (L/M), dorso/palmar (D/P), and oblique views of the MCP joint. A radiologist (L.L.), who was not involved in the evaluation of radiographs, selected 10 cases among the patients that met the inclusion criteria.

### 2.2. MCP Joint Osteophytes Scoring System

**Figure 1:**
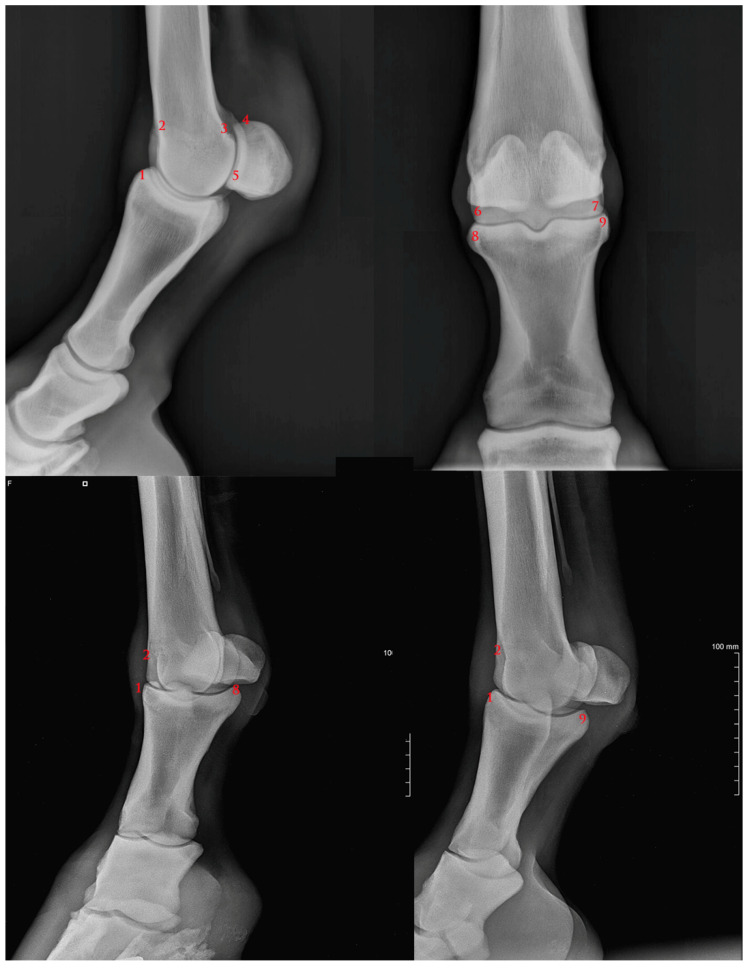
Anatomical assessment point description: **1**) P1 dorso-proximal margin; **2**) MC3 dorso-proximal margin; **3**) caudo-proximal supracondylar margin; **4**) PSB proximal margin; **5**) PSB distal margin; **6**) MC3 lateral margin; **7**) MC3 lateral margin; **8**) P1 medial margin; **9**) P1) lateral margin. P1: 1st phalanx; MC3: 3rd metacarpal bone; PSB: proximal sesamoid bones.

To assess the degree of osteophytosis it was decided to observe the radiographs and issue a judgement focusing on nine anatomical points of the MCP joint, where it is typical to find lesions characteristic of the pathology. These points are described in Figure 1.

For each anatomical point, a score was assigned with four degrees of judgement (0, 1, 2, and 3), to characterize the degree of radiographic changes in osteophytosis which corresponds to an increasing level of OA severity (none, mild, moderate, and severe, respectively). (Table 1)

### 2.3. Observers

The radiograms were examined by five veterinary medicine students (inexperienced) and four equine experienced veterinary surgeons (expert). Each observer was given an evaluation table with schematic radiographs, where the anatomical points to be examined were highlighted. Everyone worked independently on the same list of radiograms. Each observer viewed the radiograms twice, at least one week after the first observation, in a variable order, and blinded about the patient, in such a way that the observation was not influenced by the previously issued judgements.

### 2.4. Statistical analysis

The estimation of intra-observer variability established on a 95% confidence interval was defined as the minimum difference between two consecutive measurements made by the same group of observers. The intra-observer variability was based on the residue method with the differences between the measurements plotted against the average. Furthermore, the mean differences in measurements between the first and second observations for each group of observers were determined by the Bland–Altman method.

The estimate of inter-observer variability based on the 95% confidence interval was defined as the minimum difference between two consecutive measurements made by the two different groups of observers. The inter-observer variability was determined for each single evaluation point using the Pearson correlation coefficient, with the mean of the difference calculated between the two measurements. The coefficient of variability (CV) was calculated for each anatomical assessment point and for each case evaluated. The Pearson correlation coefficient was also calculated for the CV vs. the mean score assessed for each case by the two groups of observers.

All statistical calculations were performed by the statistical software MedCalc Statistical Software version 16.4.3 (MedCalc Software bvba, Ostend, Belgium; 2016).

## 3. Results

### 3.1. Population

The features of the horses included were as follows: age 2–16 years (mean age 8.20 ± 4.66 years old) and weight 453–622 kg (mean 529.4 ± 57.26 kg). Horses included three Standardbreds, two Angloarabs, two quarter horses, and three Italian saddle horses; four males, three geldings, and three mares.

### 3.2. Intra-observer Measurements

The variability between the two measurements, indicated by the mean differences between the first and second observation for each group of observers, was −2.04 ± 3.5 (mean ± standard deviation) with a 95% CI −3.04 to −1.03 for the group of inexperienced observers. For the group of expert observers, the variability between the two measurements was 0 ± 1.43 (mean ± standard deviation) with a 95% CI −0.45 to 0.45.

**Figure 2:**
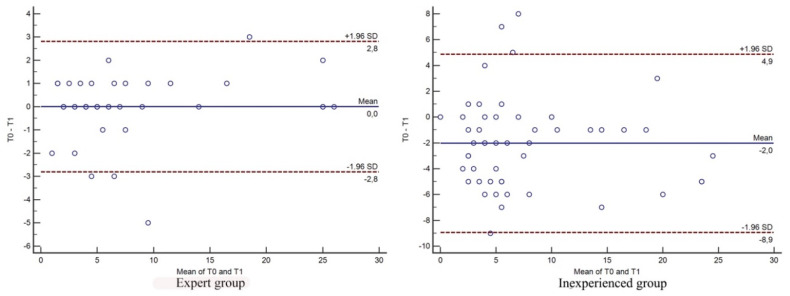
Bland–Altman plots for intra-observer measurement variability of the expert group and inexperienced group. T0 First evaluation, T1 second evaluation.

The expert group maintained values that were almost the same in the first (T0) and in the second observation (T1), with a CV of 12.77%. The inexperienced group had the least accordance and therefore the least consistency in the evaluation of the same radiograph, indicated by the standard deviation of the difference and higher CV of 37.29%. In general, the inexperienced group assigned the lowest osteophytosis scores in the first evaluation (mean 6.64 ± 0.95) and the highest in the second evaluation (8.68 ± 0.15), with the difference reaching statistical significance. The expert group at the first and second evaluations scored meanly (mean 7.82 ± 1.06). No significant differences were detected within each observed group from the first and second evaluations (Figure 2).

### 3.3. Inter-observer Measurement

The correlation was high for all observers, with r = 0.90% (*p* < 0.05). The mean difference between the observer groups was −1.13 ± 3.18 with a 95% CI −7.22 to 4.97.

The CV for measurements considering all anatomical assessment points for the inexperienced group was 66%, for the expert group was 25.07%, and for between groups was 28.85%. The mean difference between the two observations was −0.03 ± 0.29 in the expert group and 0.22 ± 0.77 in the inexperienced group. In the inexperienced group the difference was statistically significant (*p* < 0.01).

**Figure 3:**
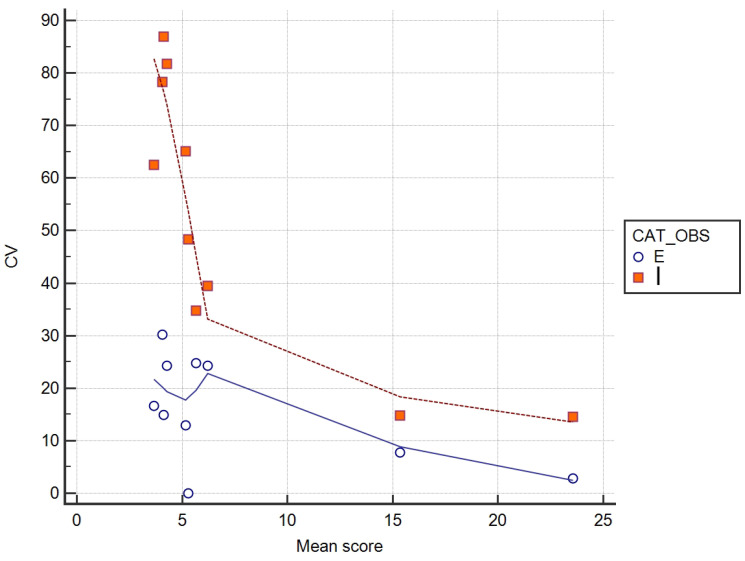
Mean score assigned for each case versus coefficient of variation (CV). Expert group (E), Inexperienced group (I).

The most relevant discordance in evaluation of assessment point was found for points 6,9,7, and 5. This finding was similar in both groups, but was significantly lower in the expert group. The CV was also evaluated versus the cases examined. The results showed that the inexperienced group had a greater CV when the score was low (low osteophytosis) (r = −0.78, *p* < 0.01) compared to the expert group, where the CV was independent of the severity of osteophytosis (r = −0.47) (Figure 3).

## 4. Discussion

The present study shows that the radiographic scoring system of MCP joint osteophytosis is a feasible method to obtain and compare OA scores with low intra- and inter-observer measurement variability. In our study, the lowest consistencies in the score are those assigned by the inexperienced group.

Several methods are currently available for routine OA monitoring. These include clinical evaluation, radiography, scintigraphy, arthroscopy, computerized tomography, and magnetic resonance [9,10,11].

In daily clinical practice, conventional radiography is still the most commonly used imaging technique [12]. Radiographic findings observed in MCP joint OA include lesions in periarticular soft tissues, synovial effusion, osteophytosis, enthesopathy, intra-articular mineralization, subchondral sclerosis, formation of subchondral cysts, and thinning of the joint space [2,7,13,14]. Although the pathophysiology of osteophyte production is not completely understood [15], it is acknowledged that they appear at the beginning of the pathological process of OA [10,14].

In the evaluation of OA evolution, osteophytes can be observed initially and before the development of joint space narrowing. [16] In human medicine, osteophytes in knee joints are important clinical findings of OA used to measure disease advancement and predict pathological manifestations. Tests carried out on cases of OA in humans have shown that osteophytes are related to the presence of pain [17]. Furthermore, studies conducted on humans and animals have shown that the radiographic evaluation of osteophytes results in good intra-observer agreement [10,11,13,14,17].

The dog stifle was scored mainly on the presence and severity of osteophytes [14]. The presence and severity of osteophytes cannot be modified using exposure control with digital radiology, which suggests the probable benefit for the use of a radiographic scoring system in the evaluation of osteophytes. This is a major radiographic feature and an important criterion for staging this disease [14,18].

For this reason, we can speculate that staging of equine OA by a radiographic osteophytosis scoring system could be a useful tool for comparing radiographs during follow-up or assessing severity of joints affected by OA.

OA scoring systems are a significant and typical tool used for the evaluation of radiographic evolution of osteoarthritis. In dogs, humans, and horses, the developed OA scoring system includes assessments of changes to surrounding soft tissues, subchondral sclerosis, and joint space width [8,14,17,19]. However, soft tissues have low contrast and are not always visible with conventional radiography [14]. Furthermore, it is not yet established empirically whether an observer can control digital radiographs to highlight soft tissue structures and how this would impact the overall evaluation of other structures. Moreover, subchondral sclerosis has been reported as the more controversial finding on radiograms, although it is more coherent with the use of MRI or CT [7]. Innes and colleagues [10] reported excellent concordant intra-observer opinions for the total score, effusion, and osteophytosis, and a good inter-observer agreement for global score, effusion, osteophytosis, and intra-articular mineralization.

To our knowledge, in the horse there are still few OA scoring systems described in the literature [3,7]. Frisbie and colleagues developed a scoring system for the equine knee, assessing the radiographs for subchondral osteolysis, enthesophytosys, and osteophyte formation. Each irregularity was graded on a scale of 0–3 for severity: 0 represented no detectable abnormality, and 3 represented the most severe change [2,3,4,5,6]. But, this scoring system considered the whole joint and not specific anatomical points. Olive and colleagues proposed a scoring system for staging OA of MCP joints of the horse, grading osteophytosis independently at 12 sites at the dorsal, palmar, lateral, and medial aspects of the metacarpal condyle and the proximal phalanx as well as at the proximal and distal aspects of each proximal sesamoid bone. Scores were attributed according to the evident dimension of the osteophytes as recommended for human OA grading [7].

In our study, we used a similar systematic approach. We considered only nine anatomical points visible on D/P, L/M, and oblique radiographic views, grading osteophytosis according to a semi-quantitative scale (graded 0–3).

In our study, the capability to replicate measurements was high for both groups of observers, which indicates the ability to consistently identify subjectively determined assessment points on radiographs, but observers with low experience needed a reference severity scale. Additionally, the high level of reproducibility for intra-observer measurements in the expert group also shows that observers can coherently reproduce the measurements for each individual radiograph.

In particular, although the variability of the intra-observer measurement was low, an insignificant variability between the first and the second observation was observed in the expert group. These results could suggest that lower levels of knowledge and experience may lead to variable application of the measurement method.

In the current study, the variability of the total inter-observer score was low. The correlation was high for all investigators, and all of them achieved the limit for statistical significance.

The variability found in the evaluations of the observers may be due to subtle variations in flexion or rotation in the positioning of the MCP joint that can modify the radiographic aspect of the anatomical points in the study required for the assessment of the severity of osteoarthritis. Therefore, accurate latero/medial and dorso/palmar-plantar radiographic views of the metacarpal/metatarsophalangeal joint and oblique views are necessary.

Further aspects could include a refining of the four-degree scale to a scale with a more precise evaluation based on the severity of the osteophytes.

Interestingly, higher variability was detected in the inexperienced group evaluating radiograph of joints affected by low grade OA, while the expert group was consistent independent of severity of disease. We suppose that students in training need to develop more skills in detecting small osteophytes, or low modification of joint profiles helped with a visual reference scale.

## 5. Conclusions

In conclusion, we recommend the scoring system proposed in our study for the following reasons. We have demonstrated a system that is easily applicable and usable by observers with different levels of knowledge, but inexperienced observers need more training and must be helped by reference images. These data are supported by the results of the low variability of inter- and intra-observer measurements in the expert group.

Therefore, the proposed scoring system appears to be a repeatable tool suitable to the radiographic scoring of the severity of MCP joint osteoarthritis.

## Figures and Tables

**Table 1:** Grading system and corresponding findings.

Grade	Severity	Findings
**0**	no osteoarthritis	normal radiographic aspect
**1**	mild osteoarthritis	slight osteophytosis and/or slight sclerosis is observed
**2**	moderate osteoarthritis	sclerosis and osteophytosis appear moderate
**3**	severe osteoarthritis	severe sclerosis and the presence of marked osteophytes
